# Supplementary material for: Comparison of Multi-locus Genotypes Detected in Aspergillus fumigatus Isolated from COVID Associated Pulmonary Aspergillosis (CAPA) and from Other Clinical and Environmental Sources
Source: J Fungi (Basel). 2023 Feb 24;9(3):298. doi: 10.3390/jof9030298 (PMC10056896; doi:10.3390/jof9030298)
Supplement: Supplementary file 1 [file jof-09-00298-s001.zip › jof-2142299-supplementary.pdf]

Table S1. Isolate characterization of the isolates analyzed in the study.

	Isolate source	Year	Sample	Source	Fragment Size (base pairs)			Number of Repeats			*cyp51A* and Promotor Mutation	AfumID Clade
					STRAf3 A	STRAf3 B	STRAf3 C	STRAf 3A	STRAf 3B	STRAf 3C		
CD7	Environment	2018	Air	Private home	129,05	176,69	80,22	7	15	6	N.A.	Susceptible
CD10	Environment	2018	Surface	Lift truck AC filter	189,21	162,26	120,84	26	10	19	N.A.	Susceptible
CD18A	Environment	2018	Surface	Taxi´s AC filter	145,34	168,05	120,51	12	12	19	N.A.	Susceptible
CD18B	Environment	2018	Surface	Taxi´s AC filter	145,47	167,97	114,57	12	12	17	N.A.	Susceptible
CD19A	Environment	2018	Surface	Taxi´s AC filter	DNA did not amplify, culture not available						-	-
CD19B	Environment	2018	Surface	Taxi´s AC filter	180,16	162,26	105,54	24	10	14	N.A.	Resistant
CD32	Environment	2018	Air	Private home	186,19	162,25	105,51	25	10	14	N.A.	Resistant
CD157	Environment	2018	Air	Bakery	Contamined, multiple peaks						-	-
CD184	Environment	2018	Air	Private home (EDC)	170,98	176,82	123,94	21	15	20	N.A.	Susceptible
CD186	Environment	2018	Air	Private home (EDC)	145,61	168,27	120,78	12	12	19	N.A.	Susceptible
CD200	Environment	2018	Air	Private home (EDC)	189,4	165,19	114,69	26	11	17	N.A.	Susceptible
CD201	Environment	2018	Air	Private home (EDC)	219,54	182,58	136,44	36	17	24	N.A.	Susceptible
CD203	Environment	2018	Air	Private home (EDC)	283,22	165,15	159,41	57	11	32	N.A.	Susceptible
CD215	Environment	2018	Air	Private home (EDC)	201,5	165,23	108,66	30	11	15	N.A.	Susceptible
CD223	Environment	2018	Air	Private home (EDC)	145,69	168,07	120,85	12	12	19	N.A.	Susceptible
CD226	Environment	2018	Air	Private home (EDC)	192,44	162,29	117,7	27	10	18	N.A.	Susceptible

CD228	Environment	2018	Air	Private home (EDC)	177,09	227,86	93,31	23	32	10	N.A.	Resistant
CD257	Environment	2018	Air	Dairy	192,33	162,25	90,02	27	10	9	N.A.	Resistant
CD259	Environment	2018	Air	Dairy	183,1	162,24	108,57	24	10	15	N.A.	Resistant
CD261	Environment	2018	Air	Dairy	252,9	167,92	156,29	47	12	31	N.A.	Susceptible
CD280	Environment	2018	Air	Private home (EDC)	216,55	159,36	177,83	35	9	38	N.A.	Resistant
HSMA34	Environment	2012	Air	H3 hematol-ogy unit	192,24	165,22	108,46	27	11	15	N.A.	Susceptible
HSMA38	Environment	2012	Air	H3 hematology unit	186,21	165,11	146,41	25	11	27	N.A.	Susceptible
HSMA44	Environment	2012	Air	H3 hematology unit	183,26	165,24	123,82	25	11	20	N.A.	Susceptible
HSMA65	Environment	2012	Air	H3 bone marrow transplant unit	195,36	165,25	102,67	28	11	13	N.A.	Susceptible
HSMA66	Environment	2012	Surface	H3 ICU unit	195,35	165,15	102,67	28	11	13	N.A.	Susceptible
HSMA67	Environment	2012	Surface	H3 hematology unit	195,34	165,15	102,67	28	11	13	N.A.	Susceptible
HSMA75	Environment	2012	Air	H3 emergency room	222,41	159,29	123,76	37	9	20	N.A.	Susceptible
HSMA91	Environment	2017	Air	H3 emergency room	189,3	162,46	114,78	26	10	17	N.A.	Susceptible
HSMA92	Environment	2017	Air	H3 emergency room	155,61	168,18	123,92	16	12	20	N.A.	Susceptible
HSMA93	Environment	2017	Surface	H3 file unit	155,56	168,02	123,88	16	12	20	N.A.	Susceptible
HSMA99	Environment	2017	Soil	H3	195,4	165,35	102,65	28	11	13	N.A.	Susceptible

				Potted plant from waiting room								
HSMA107	Environment	2017	Surface	H3 file unit	155,63	168,19	123,8	16	12	20	N.A.	Susceptible
HSMA108	Environment	2017	Soil	H3 Potted plant from waiting room	189,36	162,29	114,71	26	10	17	N.A.	Susceptible
VA7	Respiratory sample (non-COVID+)	2013	Clinical sample: Sputum	H3	237,59	162,34	80,2	42	10	6	N.A.	Resistant
VA12	Respiratory sample (non-COVID+)	2013	Clinical sample: Sputum	H3	219,56	165,27	90,01	36	11	9	N.A.	Susceptible
VA21	Respiratory sample (non-COVID+)	2013	Clinical sample: BAL	H6	219,47	171,13	120,78	36	13	19	N.A.	Susceptible
VA22	Respiratory sample (non-COVID+)	2013	Clinical sample: Bronchial secretions	H6	207,37	182,52	105,7	32	17	14	N.A.	Susceptible
VA24	Respiratory sample (non-COVID+)	2013	Clinical sample: BAL	H2	189,3	162,34	80,62	26	10	6	N.A.	Resistant
VA25	Respiratory sample (non-COVID+)	2014	Clinical sample: BAL	H7	186,28	162,13	117,59	25	10	18	N.A.	Susceptible
VA31	Respiratory sample (non-COVID+)	2014	Clinical sample: Bronchial aspirate	H11	138,76	162,34	77,16	10	10	5	N.A.	Resistant
VA52	Respiratory sample (non-COVID+)	2015	Clinical sample: Sputum	H1	142,22	162,34	102,67	11	10	13	N.A.	Resistant
VA55	Respiratory sample (non-COVID+)	2015	Clinical sample: Respira-tory secretions	H1	222,4	191,11	159,42	37	20	32	N.A.	Susceptible
VA57	Respiratory sample (non-COVID+)	2015	Clinical sample: Respira-tory secretions	H1	192,27	165,15	102,68	27	11	13	N.A.	Resistant
VA58	Respiratory sample (non-COVID+)	2015	Clinical sample: Sputum	H1	219,52	159,41	83,55	36	9	7	N.A.	Resistant
VA64	Respiratory sample (non-COVID+)	2015	Clinical sample: Respira-tory secretions	H1	186,36	165,29	123,89	26	11	20	N.A.	Susceptible
VA73	Respiratory sample (non-COVID+)	2015	Clinical sample: BAL	H1	264,97	168,07	174,69	51	12	37	N.A.	Susceptible
VA77	Deep infection (non-COVID+)	2015	Clinical sample: Bronchial biopsy	H1	174,15	171,07	127,16	22	13	21	N.A.	Susceptible

VA83	Deep infection (non-COVID+)	2015	Clinical sample: Peritoneal fluid	H1	216,43	168,22	105,65	35	12	14	N.A.	Susceptible
VA85	Respiratory sample (non-COVID+) - **R**	2015	Clinical sample: BL	H8	280,18	171	171,7	56	13	36	No amplifica-tion	Susceptible
VA86	Environment - **R**	2015	Air	Pig farming	225,38	216,58	123,78	38	28	20	G54R	Susceptible
VA95	Environment - **R**	2015	Air	N.A.	280,07	171	171,71	56	13	36	Not detected	Susceptible
VA105	Respiratory sample (non-COVID+)	2015	Clinical sample: Bronchial secretions	H2	192,38	165,27	108,75	27	11	15	N.A.	Susceptible
VA121	Respiratory sample (non-COVID+)	2016	Clinical sample: Bronchial secretions	H5	249,75	179,63	123,76	46	16	20	N.A.	Susceptible
VA132	Animal	2016	Clinical sample (animal source): Bird air sac	N.A.	204,4	165,24	102,64	31	11	13	N.A.	Susceptible
VA133	Respiratory sample (non-COVID+)	2016	Clinical sample: BAL	H11	225,54	165,27	207,84	38	11	47	N.A.	Susceptible
VA146	Respiratory sample (non-COVID+)	2017	Clinical sample: BAL	H2	152,46	165,87	102,67	15	11	13	N.A.	Susceptible
VA180	Respiratory sample (non-COVID+)	2017	Clinical sample: BAL	H8	183,27	230,85	105,61	25	33	14	N.A.	Susceptible
VA183	Deep infection (non-COVID+)	2017	Clinical sample: Chest drainage pus	Guiné	168,1	194,32	73,89	20	21	4	N.A.	Susceptible
VA219	Deep infection (non-COVID+)	2018	Clinical sample: Brain bi-opsy	H6	186,29	162,33	123,92	25	10	20	N.A.	Susceptible
VA221	Respiratory sample (non-COVID+)	2018	Clinical sample: Sputum	H1	189,47	162,5	124,01	27	10	20	N.A.	Susceptible
VA225	Respiratory sample (non-COVID+)	2018	Clinical sample: BAL, bronchial and tracheal aspi-rate	H10	180,3	165,92	83,52	24	11	7	N.A.	Susceptible
VA236	Respiratory sample (non-COVID+)	2018	Clinical sample: BL	H8	152,64	166,04	96,53	15	11	11	N.A.	Susceptible
VA268	Environment	2018	Soil	Oceanarium gravel	201,47	159,3	77,05	30	9	5	N.A.	Resistant
VA290	Animal	2018	Clinical sample (animal source): Bird air sac - diver bird	N.A.	180,23	165,81	83,39	24	11	7	N.A.	Susceptible
VA303	Respiratory sample (non-COVID+) - **R**	2018	Clinical sample: BL	H8	135,65	159,54	93,31	9	9	10	TR34/L98H	Resistant

VA304	Respiratory sample (non-COVID+)	2018	Clinical sample: BAL	H8	149,23	168,27	111,85	14	12	16	N.A.	Susceptible
VA309	Respiratory sample (non-COVID+)	2019	Clinical sample: BAL	H4	192,27	162,25	165,45	27	10	34	N.A.	Resistant
VA310	Respiratory sample (non-COVID+)	2019	Clinical sample: Sputum	H9	201,51	165,34	93,4	30	11	10	N.A.	Susceptible
VA311	Respiratory sample (non-COVID+)	2019	Clinical sample: BL	H8	186,52	165,42	123,93	26	11	20	N.A.	Susceptible
VA314	Respiratory sample (non-COVID+)	2019	Clinical sample: Respiratory sample (non-COVID+)	H5	186,39	165,32	123,99	26	11	20	N.A.	Susceptible
VA317	Deep infection (non-COVID+)	2019	Clinical sample: Brain biopsy	H1	213,35	176,61	120,81	34	15	19	N.A.	Susceptible
VA321	Respiratory sample (non-COVID+)	2019	Clinical sample: BL	H8	158,72	162,44	99,64	17	10	12	N.A.	Resistant
VA328	Respiratory sample (non-COVID+) - **R**	2019	Clinical sample: BAL	H8	213,35	162,24	86,67	34	10	8	Not detected	Resistant
VA329	Environment	2019	Hospital environment	H3	210,3	182,44	102,48	33	17	13	N.A.	Susceptible
VA330	Environment	2019	Hospital environment	H3	152,24	188,22	114,62	14	19	17	N.A.	Susceptible
VA334	Respiratory sample (non-COVID+)	2019	Clinical sample: BL	H8	198,44	182,61	130,25	29	17	22	N.A.	Susceptible
VA346	Respiratory sample (non-COVID+) - **R**	2019	Clinical sample: Bronchial secretions	H2	186,28	180	105,53	25	16	14	F46Y, M172V, N248T, D255E	Resistant
VA352	Deep infection (non-COVID+)	2019	Clinical sample: Pleural fluid	H7	DNA did not amplify, inviable, culture growth not obtained						N.A.	-
VA362	Animal	2020	Clinical sample (animal source): Granuloma from oceanarium	N.A.	129,19	165,09	143,07	7	11	26	N.A.	Susceptible
VA370	Animal	2020	Clinical sample (animal source): Strain from dolphin	N.A.	228,63	199,67	139,66	39	22	25	N.A.	Susceptible
VA378	Respiratory sample	2020	Clinical sample: Respiratory sample	H1	129,29	250,85	139,68	7	40	25	N.A.	Susceptible
VA379	Respiratory sample (COVID+)	2020	Clinical sample: Respiratory sample	H1	186,28	162,25	108,7	25	10	15	N.A.	Resistant
VA380	Respiratory sample (COVID+)	2020	Clinical sample: Respiratory sample	H1	207,47	168	120,57	32	12	19	N.A.	Susceptible

VA381	Respiratory sample (COVID+)	2020	Clinical sample: BAL	H1	204,52	159,41	90,1	31	9	9	N.A.	Resistant
VA382	Respiratory sample (COVID+)	2020	Clinical sample: Sputum	H1	135,43	162,25	90,1	9	10	9	N.A.	Resistant
VA388	Respiratory sample (COVID+)	2021	Clinical sample: Bronchial secretions	H1	192,32	162,13	120,64	27	10	19	N.A.	Susceptible
VA390	Respiratory sample (COVID+)	2021	Clinical sample: Respiratory secretions	H1	228,53	196,93	102,7	39	22	13	N.A.	Susceptible
VA392	Respiratory sample (COVID+)	2020	Clinical sample: Sputum	H1	129,22	250,87	136,54	7	40	24	N.A.	Susceptible
VA393	Animal	2021	Clinical sample (animal source): Dolphin respiratory exsudate	N.A.	129,22	165,24	150	7	11	29	N.A.	Susceptible
VA394	Respiratory sample (COVID+)	2021	Clinical sample: Respiratory secretions	H1	195,33	162,38	120,79	28	10	19	N.A.	Susceptible
VA396	Respiratory sample	2021	Clinical sample: BAL	H1	186,16	165,09	120,81	25	11	19	N.A.	Susceptible
VA399	Respiratory sample (COVID+)	2021	Clinical sample: BAL	H5	180,07	162,25	105,49	23	10	14	N.A.	Resistant
VA404	Respiratory sample (non-COVID+)	2021	Clinical sample: Respiratory secretions	H2	240,58	165,15	177,75	43	11	38	N.A.	Susceptible
VA420	Environment	2021	Soil	Beach	DNA did not amplify, inviable, culture growth not obtained							-
VA426	Respiratory sample (non-COVID+)	2021	Clinical sample: Bronchial secretions	H2	204,29	168,14	93,27	31	12	10	N.A.	Resistant
VA429	Respiratory sample (COVID+)	2021	Clinical sample: Respiratory secretions	H1	258,84	165,05	93,03	49	11	10	N.A.	Susceptible
VA437	Respiratory sample (non-COVID+)	2021	Clinical sample: Respiratory secretions	H2	189,38	165,33	123,95	26	11	20	N.A.	Susceptible
VA442	Environment	2021	Soil sample: Beach sand	N.A.	145,62	168,02	120,79	12	12	19	N.A.	Susceptible
VA443	Respiratory sample (COVID+)	2021	Clinical sample: Respiratory secretions	H1	155,49	159,41	93,37	16	9	10	N.A.	Resistant
VA449	Respiratory sample (non-COVID+)	2022	Clinical sample: Bronchial secretions	N.A.	152,39	273,27	114,59	15	47	17	N.A.	Susceptible
VA458	Environment	2022	Water	Water reservoir	155,45	162,2	129,88	16	10	22	N.A.	Susceptible
VA459	Animal	2022	Clinical sample (animal source): Dolphin respiratory exudate	N.A.	204,42	159,27	80,14	31	9	6	N.A.	Resistant

VA299CP	Environment - **R**	2019	Air sample: Cowshed	N.A.	201,47	159,29	89,81	30	9	9	TR34/L98H/N 248K	Resistant
V A873CP	Environment - **R**	2019	Filter from worker's mask	Waste sorting industry	One of the markers did not amplify						TR34/L98H/N 248K	-
VA1209CP	Environment - **R**	2019	Filter from worker's mask	Waste sorting industry	One of the markers did not amplify						*Wild type*	-

AC – air conditioner; EDC – electrostatic dust collector; BAL – bronchoalveolar lavage; BL – bronchial lavage; H1 to H10 –hospitals' ID (from where the isolates were collected); N.A. – non applicable; R – resistant to azoles.

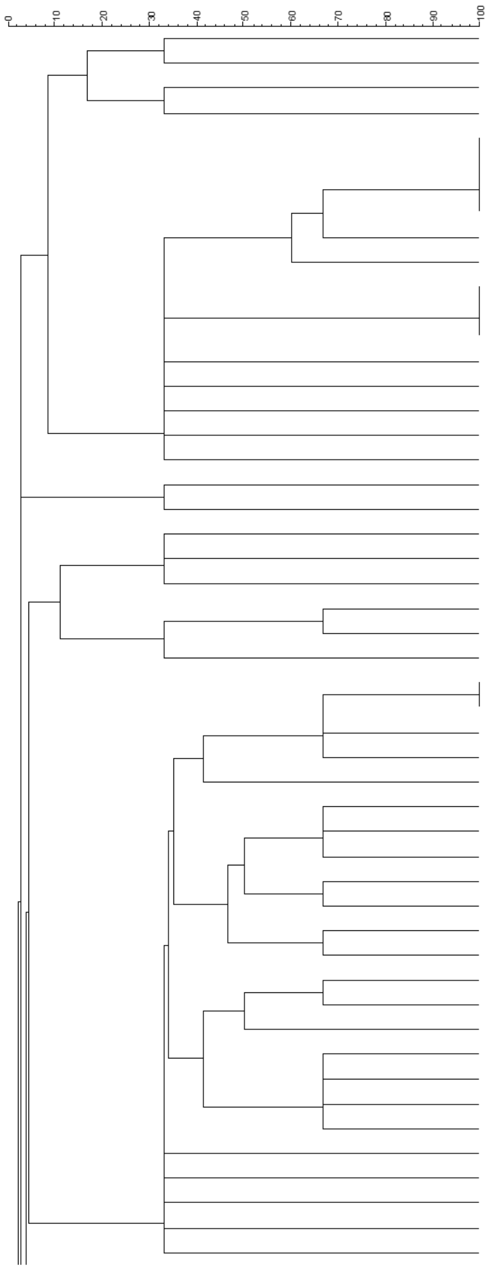

STRAf 3A	STRAf 3B	STRAf 3C	Source	Isolate
38	28	20	Environmental	VA86 - **R**
46	16	20	Respiratory samples (non-COVID+)	VA121
34	15	19	Deep infection (non-COVID+)	VA317
21	15	20	Environmental	CD184
12	12	19	Environmental	VA442
12	12	19	Environmental	CD186
12	12	19	Environmental	CD223
12	12	19	Environmental	CD18A
32	12	19	Respiratory samples (COVID+)	VA380
12	12	17	Environmental	CD18B
16	12	20	Environmental	HSMA92
16	12	20	Environmental	HSMA93
16	12	20	Environmental	HSMA107
35	12	14	Deep infection (non-COVID+)	VA83
47	12	31	Environmental	CD261
51	12	37	Respiratory samples (non-COVID+)	VA73
14	12	16	Respiratory samples (non-COVID+)	VA304
31	12	10	Respiratory samples (non-COVID+)	VA426
14	19	17	Environmental	VA330
15	47	17	Respiratory samples (non-COVID+)	VA449
33	17	13	Environmental	VA329
29	17	22	Respiratory samples (non-COVID+)	VA334
36	17	24	Environmental	CD201
25	16	14	Respiratory samples (non-COVID+)	VA346 - **R**
25	33	14	Respiratory samples (non-COVID+)	VA180
32	17	14	Respiratory samples (non-COVID+)	VA22
26	10	17	Environmental	HSMA91
26	10	17	Environmental	HSMA108
26	10	6	Respiratory samples (non-COVID+)	VA24
26	10	19	Environmental	CD10
42	10	6	Respiratory samples (non-COVID+)	VA7
27	10	18	Environmental	CD226
27	10	34	Respiratory samples (non-COVID+)	VA309
27	10	20	Respiratory samples (non-COVID+)	VA221
27	10	19	Respiratory samples (COVID+)	VA388
28	10	19	Respiratory samples (COVID+)	VA394
9	10	9	Respiratory samples (COVID+)	VA382
27	10	9	Environmental	CD257
23	10	14	Respiratory samples (COVID+)	VA399
24	10	14	Environmental	CD19B
24	10	15	Environmental	CD259
25	10	15	Respiratory samples (COVID+)	VA379
25	10	20	Deep infection (non-COVID+)	VA219
25	10	14	Environmental	CD32
25	10	18	Respiratory samples (non-COVID+)	VA25
34	10	8	Respiratory samples (non-COVID+)	VA328 - **R**
16	10	22	Environmental	VA458
10	10	5	Respiratory samples (non-COVID+)	VA31
11	10	13	Respiratory samples (non-COVID+)	VA52
17	10	12	Respiratory samples (non-COVID+)	VA321

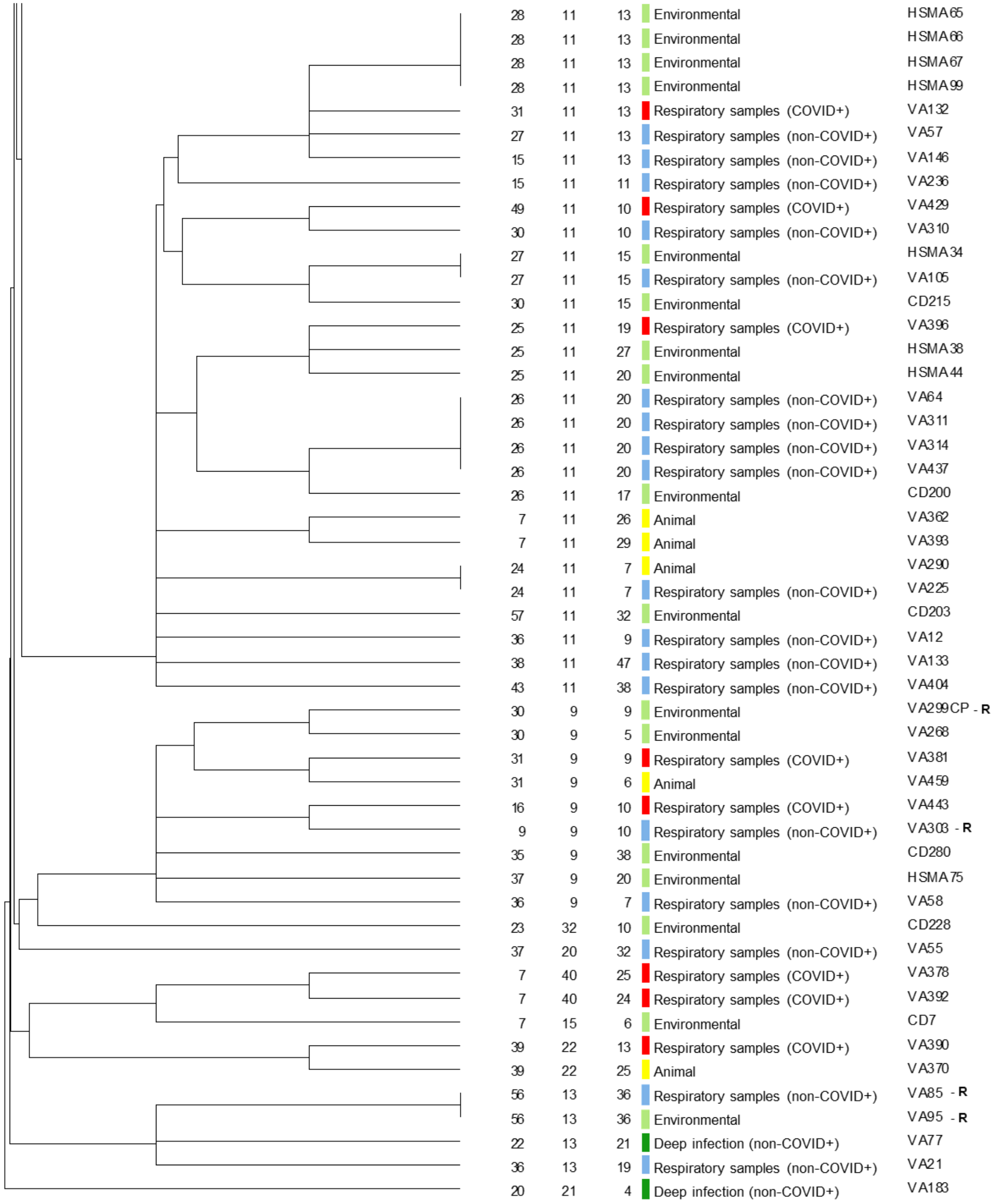

Figure S1. Dendrogram obtained in the Bionumerics 6.6 software (Applied Maths) using a categorical similarity coefficient and an unweighted paired group method with arithmetic mean (UPGMA) for the cluster analysis. R – resistant to azoles.
